# Association of Phosphorylated Tau Biomarkers With Amyloid Positron Emission Tomography vs Tau Positron Emission Tomography

**DOI:** 10.1001/jamaneurol.2022.4485

**Published:** 2022-12-12

**Authors:** Joseph Therriault, Marie Vermeiren, Stijn Servaes, Cécile Tissot, Nicholas J. Ashton, Andréa Lessa Benedet, Thomas K. Karikari, Juan Lantero-Rodriguez, Wagner S. Brum, Firoza Z. Lussier, Gleb Bezgin, Jenna Stevenson, Nesrine Rahmouni, Peter Kunach, Yi-Ting Wang, Jaime Fernandez-Arias, Kely Quispialaya Socualaya, Arthur C. Macedo, João Pedro Ferrari-Souza, Pâmela C. L. Ferreira, Bruna Bellaver, Douglas T. Leffa, Eduardo R. Zimmer, Paolo Vitali, Jean-Paul Soucy, Gallen Triana-Baltzer, Hartmuth C. Kolb, Tharick A. Pascoal, Paramita Saha-Chaudhuri, Serge Gauthier, Henrik Zetterberg, Kaj Blennow, Pedro Rosa-Neto

**Affiliations:** 1 Translational Neuroimaging Laboratory, McGill Research Centre for Studies in Aging, Montreal, Quebec, Canada; 2 Department of Neurology and Neurosurgery, Faculty of Medicine, McGill University, Montreal, Quebec, Canada; 3 Erasmus Medical Center, Erasmus University Rotterdam, Rotterdam, the Netherlands; 4 Department of Psychiatry and Neurochemistry, Institute of Neuroscience and Physiology, The Sahlgrenska Academy, University of Gothenburg, Mölndal, Sweden; 5 Wallenberg Centre for Molecular Medicine, University of Gothenburg, Gothenburg, Sweden; 6 King’s College London, Institute of Psychiatry, Psychology and Neuroscience, Maurice Wohl Institute Clinical Neuroscience Institute, London, United Kingdom; 7 NIHR Biomedical Research Centre for Mental Health and Biomedical Research Unit for Dementia at South London and Maudsley NHS Foundation, London, United Kingdom; 8 Department of Neurology and Psychiatry, University of Pittsburgh School of Medicine, Pittsburgh, Pennsylvania; 9 Department of Pharmacology, Graduate Program in Biological Sciences: Biochemistry and Pharmacology and Therapeutics, Universidade Federal do Rio Grande do Sul, Porto Alegre, Brazil; 10 Neuroscience Biomarkers, Janssen Research & Development, La Jolla, California; 11 Department of Math & Statistics, University of Vermont, Burlington; 12 Clinical Neurochemistry Laboratory, Sahlgrenska University Hospital, Mölndal, Sweden; 13 Department of Neurodegenerative Disease, UCL Institute of Neurology, Queen Square, London, United Kingdom; 14 UK Dementia Research Institute at UCL, London, United Kingdom; 15 Hong Kong Center for Neurodegenerative Diseases, Clear Water Bay, Hong Kong, China

## Abstract

**Importance:**

The recent proliferation of phosphorylated tau (p-tau) biomarkers has raised questions about their preferential association with the hallmark pathologies of Alzheimer disease (AD): amyloid-β plaques and tau neurofibrillary tangles.

**Objective:**

To determine whether cerebrospinal fluid (CSF) and plasma p-tau biomarkers preferentially reflect cerebral β-amyloidosis or neurofibrillary tangle aggregation measured with positron emission tomography (PET).

**Design, Setting, and Participants:**

This was a cross-sectional study of 2 observational cohorts: the Translational Biomarkers in Aging and Dementia (TRIAD) study, with data collected between October 2017 and August 2021, and the Alzheimer’s Disease Neuroimaging Initiative (ADNI), with data collected between September 2015 and November 2019. TRIAD was a single-center study, and ADNI was a multicenter study. Two independent subsamples were derived from TRIAD. The first TRIAD subsample comprised individuals assessed with CSF p-tau (p-tau_181_, p-tau_217_, p-tau_231_, p-tau_235_), [^18^F]AZD4694 amyloid PET, and [^18^F]MK6240 tau PET. The second TRIAD subsample included individuals assessed with plasma p-tau (p-tau_181_, p-tau_217_, p-tau_231_), [^18^F]AZD4694 amyloid PET, and [^18^F]MK6240 tau PET. An independent cohort from ADNI comprised individuals assessed with CSF p-tau_181_, [^18^F]florbetapir PET, and [^18^F]flortaucipir PET. Participants were included based on the availability of p-tau and PET biomarker assessments collected within 9 months of each other. Exclusion criteria were a history of head trauma or magnetic resonance imaging/PET safety contraindications. No participants who met eligibility criteria were excluded.

**Exposures:**

Amyloid PET, tau PET, and CSF and plasma assessments of p-tau measured with single molecule array (Simoa) assay or enzyme-linked immunosorbent assay.

**Main Outcomes and Measures:**

Associations between p-tau biomarkers with amyloid PET and tau PET.

**Results:**

A total of 609 participants (mean [SD] age, 66.9 [13.6] years; 347 female [57%]; 262 male [43%]) were included in the study. For all 4 phosphorylation sites assessed in CSF, p-tau was significantly more closely associated with amyloid-PET values than tau-PET values (p-tau_181_ difference, 13%; 95% CI, 3%-22%; *P* = .006; p-tau_217_ difference, 11%; 95% CI, 3%-20%; *P* = .003; p-tau_231_ difference, 15%; 95% CI, 5%-22%; *P* < .001; p-tau_235_ difference, 9%; 95% CI, 1%-19%; *P* = .02) . These results were replicated with plasma p-tau_181_ (difference, 11%; 95% CI, 1%-22%; *P* = .02), p-tau_217_ (difference, 9%; 95% CI, 1%-19%; *P* = .02), p-tau_231_ (difference, 13%; 95% CI, 3%-24%; *P* = .009), and CSF p-tau_181_ (difference, 9%; 95% CI, 1%-21%; *P* = .02) in independent cohorts.

**Conclusions and Relevance:**

Results of this cross-sectional study of 2 observational cohorts suggest that the p-tau abnormality as an early event in AD pathogenesis was associated with amyloid-β accumulation and highlights the need for careful interpretation of p-tau biomarkers in the context of the amyloid/tau/neurodegeneration, or A/T/(N), framework.

## Key Points

**Question:**

Do soluble phosphorylated tau (p-tau) biomarkers preferentially reflect the presence of cerebral β-amyloidosis or tau neurofibrillary tangle aggregation?

**Findings:**

In this cross-sectional study of 2 observational cohorts, 4 p-tau biomarkers in the cerebrospinal fluid (CSF; p-tau_181_, p-tau_217_, p-tau_231_, p-tau_235_) were significantly more closely associated with amyloid PET (positron emission tomography) than with tau PET. These results were replicated in an independent group of individuals with plasma p-tau_181_, p-tau_217_, and p-tau_231_ and in another independent cohort with CSF p-tau_181_.

**Meaning:**

Results suggest that soluble p-tau biomarkers are more closely associated with cerebral amyloid-β than with tau aggregation assessed with PET; this finding supports the need for careful interpretation of p-tau biomarkers in the context of the amyloid/tau/neurodegeneration, or A/T/(N), framework.

## Teaser

This cross-sectional study of 2 observational cohorts investigates if phosphorylated tau biomarkers are more associated with the presence of cerebral β-amyloidosis or tau neurofibrillary tangle aggregation.

## Introduction

Alzheimer disease (AD) is defined by the presence of cerebral amyloid-β plaques and tau neurofibrillary tangles.^1,2^ The A/T/(N) biomarker classification system identifies 3 classes of AD biomarkers: amyloid-β, tau, and neurodegeneration, in which amyloid-β and tau biomarkers are specific to AD.^3,4^ Amyloid-β biomarkers include amyloid positron emission tomography (PET) as well as cerebrospinal fluid (CSF) and plasma concentrations of amyloid-β. Tau biomarkers include quantification of insoluble neurofibrillary tangles using PET, as well as soluble phosphorylated tau (p-tau) in the CSF and plasma. Because of their specificity, amyloid-β and tau biomarkers are increasingly used in AD diagnosis^5^ and as inclusion criteria for disease-modifying clinical trials.^6,7^

Although soluble p-tau biomarkers are interpreted as biomarkers of tau pathology, several recent observational studies provide evidence that concentrations of p-tau are closely correlated with amyloid-β deposition.^8,9,10,11,12,13,14^ A longitudinal study in autosomal dominant AD provides evidence that concentrations of soluble p-tau biomarkers begin to rise in conjunction with amyloid-β aggregation, several years before neocortical tau abnormality.^15^ Furthermore, longitudinal biomarker studies in sporadic AD report that soluble p-tau_217_ mediates the association between amyloid-β and tau-PET change.^8,16^ Correspondingly, recent biomarker models of AD suggest that p-tau reflects a state between amyloid-β plaques and tau aggregation.^16,17,18^ However, it is unclear to what extent biofluid measurements of p-tau are preferentially associated with the presence of amyloid-β or tau neurofibrillary tangles in the brain.

The objective of the current study was to determine whether soluble p-tau biomarkers are preferentially associated with cerebral amyloid-β plaques or tau neurofibrillary tangles. We evaluated the association between 4 p-tau biomarkers in the CSF (p-tau_181_, p-tau_217_, p-tau_231_, p-tau_235_) and 3 in plasma (p-tau_181_, p-tau_217_, p-tau_231_) with amyloid-β and tau aggregation assessed with PET in the Translational Biomarkers in Aging and Dementia (TRIAD) study cohort. In addition, we evaluated the association between CSF p-tau_181_ with amyloid PET and tau PET in the Alzheimer Disease Neuroimaging Initiative (ADNI) cohort.

## Methods

### Participants

#### Translational Biomarkers in Aging and Dementia

This study was approved by the Montreal Neurological Institute PET working committee and the Douglas Mental Health University Institute Research Ethics Board. Written informed consent was obtained for all participants. For this cross-sectional study, we assessed 2 independent subsamples of participants in the TRIAD^19^ cohort: a CSF p-tau subsample (n = 181) and a plasma p-tau subsample (n = 171). Participants included in the CSF subsample had measures of CSF p-tau (p-tau_181_, p-tau_217_, p-tau_231_, p-tau_235_), amyloid PET with [^18^F]AZD4694, tau PET with [^18^F]MK6240, and magnetic resonance imaging (MRI). The median (IQR) time difference between CSF and PET data collection was 53 (86) days. In the second subsample (n = 171), individuals had measures of plasma p-tau (p-tau_181_, p-tau_217_, p-tau_231_), amyloid PET with [^18^F]AZD4694, tau PET with [^18^F]MK6240, and MRI. The median (IQR) time difference between plasma and PET data collection was 70 (112) days. The individuals in the second TRIAD subsample did not have CSF measures of p-tau and thus represent an independent sample of individuals. Participants had paired fluid p-tau and PET biomarker assessments available within a 9-month interval. All individuals were included between October 2017 and August 2021. Individuals from the following race and ethnicity categories were included: Asian, Black, Hawaiian/Pacific Islander, Hispanic/Latinx, non-Hispanic/Latinx, multiracial, White, and unknown/not reported. Race and ethnicity were identified using official National Institutes of Health classifications. This study followed the Strengthening the Reporting of Observational Studies in Epidemiology (STROBE) reporting guidelines.

Cognitively unimpaired (CU) individuals had no objective cognitive impairment and a Clinical Dementia Rating (CDR) score of 0. Individuals with mild cognitive impairment had subjective and/or objective cognitive impairment and a CDR score of 0.5.^20^ Individuals with dementia had a CDR score of 1 or 2.^21^ Participants were excluded from this study if they had systemic conditions that were not adequately controlled through a stable medication regimen. Other exclusion criteria were active substance abuse, recent head trauma, recent major surgery, or MRI/PET safety contraindications. PET acquisition and processing are described in eMethods 1 of the Supplement. All p-tau residues measured from the CSF, as well as plasma p-tau_181_ and p-tau_231_, were quantified in the Clinical Neurochemistry Laboratory, University of Gothenburg, Mölndal, Sweden, by scientists blinded to participant clinical and PET information; this information is described in detail in eMethods 2 of Supplement. Plasma p-tau_217_ was quantified by scientists at Janssen Research & Development blinded to clinical and PET information.

#### Alzheimer Disease Neuroimaging Initiative

The ADNI study was approved by the institutional review boards of all participating institutions. All participants provided informed written consent. We examined the open-access ADNI cohort, a North American multisite cohort launched in 2003. All participants had amyloid PET with [^18^F]florbetapir, tau-PET with [^18^F]flortaucipir, and CSF p-tau_181_. ADNI PET acquisition and processing data are described in eMethods 1 of the Supplement, and CSF p-tau_181_ quantification is described in eMethods 2 of the Supplement. The median (IQR) time difference between CSF and PET data collection was 13 (29) days. CU participants had a CDR of 0, individuals with mild cognitive impairment had a CDR of 0.5, and individuals with dementia had a CDR score of 1 or 2. Full information regarding the ADNI inclusion and exclusion criteria is available on the ADNI informational site.^22^ Plasma p-tau_181_ was not investigated in ADNI due to the small number of individuals with plasma p-tau evaluations and tau PET at the same visit.

### Statistical Analysis

Statistical analyses were performed in R, version 4.1.1 (R Foundation for Statistical Computing) and Matlab, version 2015a (MathWorks). Assumptions of normality were tested using the D’Agastino-Pearson normality test. Associations between p-tau biomarkers with [^18^F]AZD4694 PET and [^18^F]MK6240 PET were investigated using the Spearman nonparametric test. Statistical evaluation of whether correlations were significantly different was performed in R using the cocor package,^23^ a statistical framework for comparing associations between intercorrelated measurements. As secondary confirmatory analyses, we conducted partial correlation analyses to determine the extent to which p-tau biomarker concentrations were associated with amyloid PET when correcting for tau PET and tau PET correcting for amyloid PET using the ppcor package. We also conducted analyses correcting for age and sex. *P *values were 2-sided, and statistical significance was defined as *P* <.05.

Because PET measures of pathology reflect accumulation within specific brain regions, which may preferentially reflect protein aggregation at specific disease stages, we conducted supplementary sensitivity analyses stratified by cognitive impairment, and we used summary composite regions of interest (ROIs) considered to become positive earlier in the AD process. For amyloid PET, the Biofinder Early Aβ-PET ROI^24^ was used, and for tau PET, the inferior temporal cortical ROI was used, previously implemented to capture early tau aggregation in studies of CU individuals^25,26^ and in the early stages of autosomal dominant AD.^27^ We also compared global amyloid PET with tau-PET uptake in Braak I-II regions. We also compared CSF concentrations of Aβ (indexed by the Aβ42/40 ratio) with tau-PET uptake. Finally, we compared whole-cortex amyloid-PET and whole-cortex tau-PET standardized uptake value ratios (SUVRs).

## Results

### Participants

A total of 609 participants (mean [SD] age, 66.9 [13.6] years; 347 female [57%]; 262 male [43%]) were included in the study. The first TRIAD subsample included 181 participants; the second subsample included 171 participants. The mean (SD) age of participants in the CSF TRIAD subsample was 61.7 (17.9) years, with 196 female individuals (55.7%) and 156 male individuals (44.3%). The mean (SD) age of participants in the plasma TRIAD subsample was 66.3 (15.2) years, with 113 female individuals (66.1%) and 58 male individuals (33.9%). The ADNI cohort included a total of 257 participants (mean [SD] age, 70.6 [6.7] years; 131 female [51.0%]; 126 male [49.0%]). Our study included the following race and ethnicity groups: 17 Asian (2.8%), 17 Black (2.8%), 1 Hawaiian/Pacific Islander (0.2%), 10 Hispanic/Latinx (1.6%), 577 non-Hispanic/Latinx (94.7%), 4 multiracial (0.7%), 551 White (90.5%), and 19 unknown/not reported (3.1%). Demographic, clinical, and biomarker information for all samples is reported in the Table.

**Table.** Demographic Characteristics of the Samples

Characteristic	**No. (%)**			
	Young adults	Cognitively unimpaired older adults	Mild cognitive impairment	Dementia
**A: TRIAD cerebrospinal fluid sample**				
No.	27	86	45	23
Age, mean (SD), y	22.8 (1.9)	69.5 (8.0)	71.3 (7.5)	62.4 (6.5)
Sex				
Male	11 (40.7)	32 (37.2)	18 (40.0)	12 (52.2)
Female	16 (59.3)	54 (62.8)	27 (60.0)	11 (47.8)
Education, mean (SD), y	16.7 (1.5)	14.8 (3.5)	14.9 (3.2)	15.1 (3.5)
APOE ε4 carriers, %	6 (22.2)	25 (29.1)	17 (37.7)	13 (56.5)
MMSE, mean (SD)	29.8 (0.5)	29.1 (1.0)	27.7 (1.8)	18.7 (5.6)
Self-reported race				
American Indian/Alaskan Native	0 (0)	0 (0)	0 (0)	0 (0)
Asian	9 (33.3)	0 (0)	1 (2.2)	1 (4.3)
Black	1 (3.7)	1 (1.2)	0 (2.3)	0 (0)
Hawaiian/Pacific Islander	0 (0)	0 (0)	0 (0)	0 (0)
Multiracial	1 (3.7)	0 (0)	0 (0)	0 (0)
White	16 (59.3)	78 (90.7)	42 (93.3)	21 (91.3)
Unknown/not reported	0 (0)	7 (8.1)	2 (4.4)	1 (4.3)
Self-reported ethnicity				
Hispanic/Latinx	0 (0)	1 (1.2)	0 (0)	0 (0)
Not Hispanic/Latinx	27 (100)	78 (90.7)	43 (95.5)	21 (91.3)
Unknown/not reported	0 (0)	7 (8.1)	2 (4.4)	2 (8.7)
**B: TRIAD plasma sample**				
No.	9	88	43	31
Age, mean (SD), y	22.6 (1.6)	69.5 (12.2)	67.2 (11.4)	69.0 (9.7)
Sex				
Male	2 (22.2)	27 (30.7)	17 (39.5)	12 (38.7)
Female	7 (77.8)	61 (69.3)	26 (60.5)	19 (61.3)
Education, mean (SD), y	16.1 (1.4)	15.9 (4.0)	15.2 (4.1)	13.8 (3.6)
APOE ε4 carriers, %	3 (33.3)	19 (21.6)	15 (38.5)	15 (48.3)
MMSE, mean (SD)	30.0 (0.0)	29.2 (1.1)	28.0 (1.8)	20.0 (5.9)
Self-reported race				
American Indian/Alaskan Native	0 (0)	0 (0)	0 (0)	0 (0)
Asian	4 (44.4)	0 (0)	1 (2.3)	1 (3.2)
Black	0 (0)	1 (1.1)	1 (2.3)	0 (0)
Hawaiian/Pacific Islander	0 (0)	0 (0)	0 (0)	0 (0)
Multiracial	0 (0)	0 (0)	0 (0)	0 (0)
White	5 (55.6)	80 (90.9)	41 (95.3)	28 (90.3)
Unknown/not reported	0 (0)	7 (7.9)	0 (0)	2 (6.5)
Self-reported ethnicity				
Hispanic/Latinx	0 (0)	1 (1.1)	0 (0)	0 (0)
Not Hispanic/Latinx	9 (100)	80 (90.1)	43 (100)	29 (93.5)
Unknown/not reported	0 (0)	7 (7.8)	0 (0)	2 (6.5)
**C: ADNI CSF sample**				
No.	0	153	88	16
Age, mean (SD), y	NA	71.4 (6.3)	69.7 (7.1)	67.9 (8.0)
Sex, No. (%)				
Male	NA	63 (41.2)	54 (61.4)	9 (56.2)
Female		90 (58.8)	34 (38.6)	7 (43.8)
Education, mean (SD), y	NA	16.8 (2.4)	16.6 (2.6)	15.4 (2.2)
*APOE *ε4 carriers, %	NA	50 (32.9)	34 (41.5)	11 (68.8)
MMSE, mean (SD)	NA	29.1 (1.2)	27.9 (2.0)	20.9 (2.8)
Self-reported race, No. (%)				
American Indian/Alaska Native	NA	0 (0)	0 (0)	0 (0)
Asian		0 (0)	0 (0)	0 (0)
Black		10 (6.5)	2 (2.3)	1 (6.3)
Hawaiian/Pacific Islander		0 (0)	1 (1.1)	0 (0)
Multiracial		3 (2.0)	0 (0)	0 (0)
White		140 (91.5)	85 (96.6)	15 (93.7)
Unknown/not reported		0 (0)	0 (0)	0 (0)
Self-reported ethnicity, No. (%)				
Hispanic/Latinx	NA	6 (3.9)	2 (2.3)	0 (0)
Not Hispanic/Latinx		145 (94.8)	86 (97.7)	16 (100)
Unknown/not reported		2 (1.3)	0 (0)	0 (0)

Abbreviations: ADNI, Alzheimer’s Disease Neuroimaging Initiative; APOE, apolipoprotein E; CSF, cerebrospinal fluid; MMSE, Mini-Mental State Examination; NA, not applicable; SUVR, standardized uptake value ratio; TRIAD, the Translational Biomarkers in Aging and Dementia.

### Associations Between CSF p-Tau and PET Biomarkers

**Figure 1.**
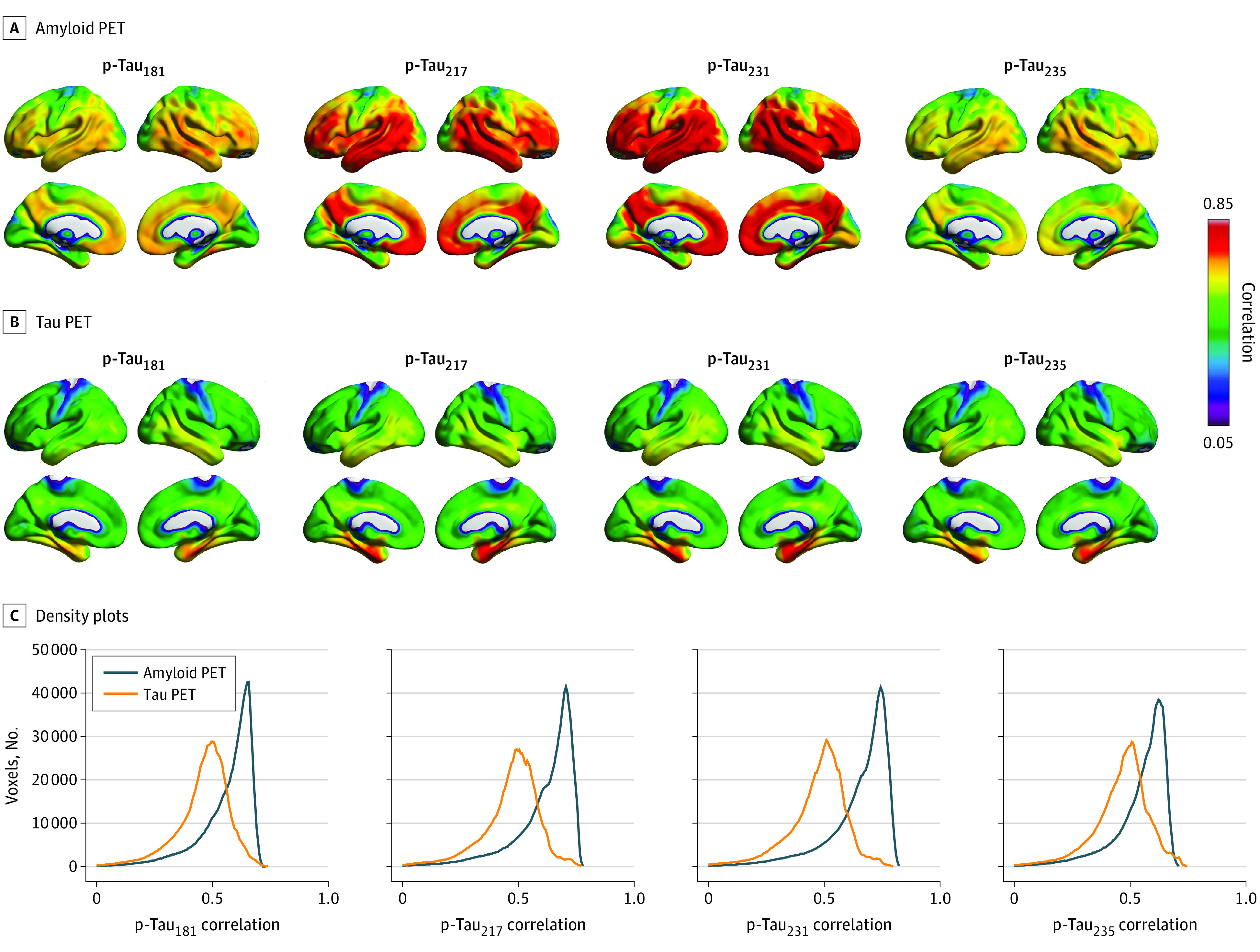
Association of Cerebrospinal Fluid (CSF) Phosphorylated Tau (p-Tau) Biomarkers With Amyloid Positron Emission Tomography (PET) and Tau PET Across the Cerebral Cortex Brain images show the distribution of associations between CSF p-tau biomarkers (p-tau_181_, p-tau_217_, p-tau_231_, and p-tau_235_) and [^18^F]AZD4694 amyloid PET (A) and [^18^F]MK6240 tau PET (B). C, Density plots depict the magnitude and frequency of the correlations in voxels per CSF p-tau epitope and imaging biomarker. For all CSF p-tau phosphorylation sites, most voxels had correlation values with amyloid PET between 0.65 and 0.75. In contrast, the majority of voxels had correlations around 0.50 with tau PET, with limited numbers of voxels having correlations between 0.65 and 0.75.

Figure 1 displays voxelwise associations between CSF p-tau_181_, p-tau_217_, p-tau_231_, and p-tau_235_ with amyloid PET and with tau PET in the TRIAD cohort. CSF p-tau_231_ and p-tau_217_ had the strongest associations with amyloid PET across the cerebral cortex. Lower correlations were observed across the cerebral cortex for CSF p-tau_181_, p-tau_217_, p-tau_231_, and p-tau_235_ with tau PET. For all CSF p-tau phosphorylation sites, correlations above 0.65 were restricted to the medial temporal cortices. Frequency distributions of correlations are displayed in Figure 1C. For all p-tau biomarkers, associations with amyloid PET were more widespread across the brain.

**Figure 2.**
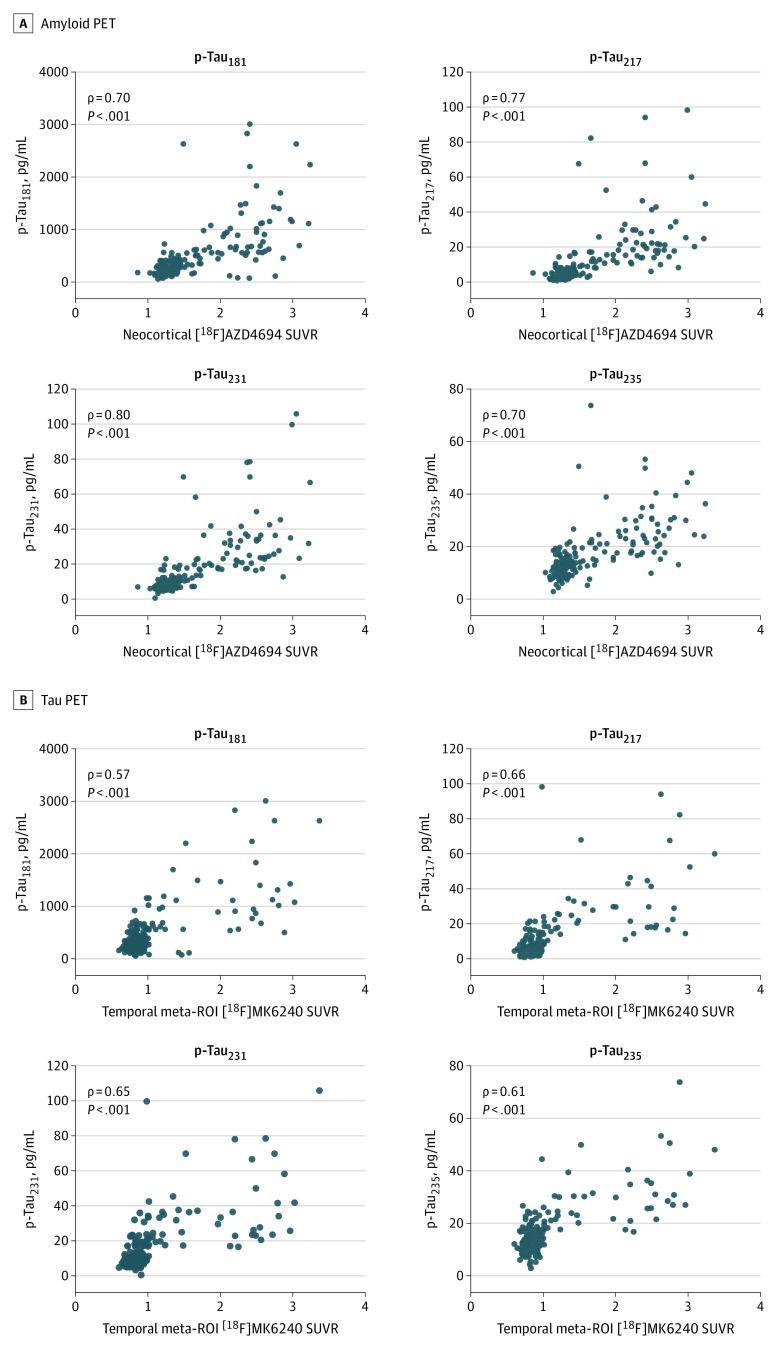
Association of Cerebrospinal Fluid (CSF) Phosphorylated Tau (p-Tau) Biomarkers With Summary Amyloid Positron Emission Tomography (PET) and Tau PET Outcomes Scatterplots show the association between CSF p-tau_181_, p-tau_217_, p-tau_231_, p-tau_235_, and summary measures of amyloid PET and tau PET in the Translational Biomarkers in Aging and Dementia study. ROI indicates region of interest; SUVR, standardized uptake value ratio.

**Figure 3.**
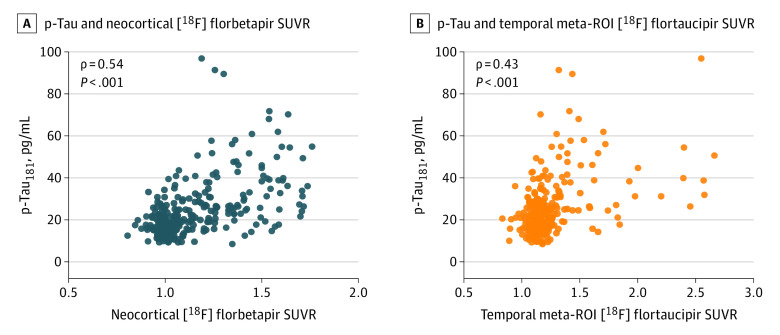
Association of Cerebrospinal Fluid (CSF) Phosphorylated Tau (p-Tau)_181_ Biomarkers With Summary Amyloid Positron Emission Tomography (PET) and Tau PET in the Alzheimer’s Disease Neuroimaging Initiative Scatterplots show the association between CSF p-tau_181_ and summary measures of [^18^F]florbetapir amyloid PET and [^18^F]flortaucipir tau PET in the Alzheimer Disease Neuroimaging Initiative study. ROI indicates region of interest; SUVR, standardized uptake value ratio.

Figure 2 displays associations between CSF p-tau biomarkers with summary measures of amyloid PET and tau PET in the TRIAD cohort. CSF concentrations of p-tau_217_ and p-tau_231_ were the most closely associated with neocortical summary measurements of amyloid PET (p-tau_217_, ρ = 0.77; 95% CI, 0.69-0.82; *P* < .001; p-tau_231_, ρ = 0.80; 95% CI, 0.73-0.85; *P* < .001). Correlations for CSF p-tau_181_ and p-tau_235_ with summary amyloid-PET SUVR were relatively lower (p-tau_181_, ρ = 0.70; 95% CI, 0.61-0.77; *P* < .001; p-tau_235_, ρ = 0.70; 95% CI, 0.60-0.76; *P* < .001). When investigating associations between CSF p-tau phosphorylation and tau-PET summary measurements, we observed that p-tau_217_ and p-tau_231_ biomarkers were most closely associated with tau PET in the temporal meta-ROI (p-tau_217_, ρ = 0.66; 95% CI, 0.57-0.74; *P* < .001; p-tau_231_, ρ = 0.65; 95% CI, 0.56-0.73; *P* < .001). Lower correlations were observed for p-tau_181_ and p-tau_235_ (p-tau_181_, ρ = 0.57; 95% CI, 0.46-0.66; *P* < .001; p-tau_235_, ρ = 0.61; 95% CI, 0.50-0.70; *P* < .001). Comparison of correlations revealed that for all p-tau phosphorylation sites, p-tau was significantly more closely associated with summary measurements of amyloid PET than with summary measures of tau-PET (p-tau_181_ difference, 13%; *t* value = 2.54; *P* = .006; 95% CI, 0.03-0.22; p-tau_217_ difference, 11%; *t* value = 2.77; *P* = .003; 95% CI, 0.03-0.20; p-tau_231_ difference, 15%; *t* value = 3.96; *P* < .001; 95% CI, 0.05-0.22; p-tau_235_ difference, 9%; *t* value = 1.98; 95% CI, 0.01-0.19; *P* = .02). In sensitivity analyses using the early amyloid PET from the Biomarkers for Identifying Neurodegenerative Disorders Early and Reliably (BioFINDER) study and inferior temporal ROIs, all p-tau biomarkers were more closely associated with amyloid PET than tau PET (eResults, eFigure 1, and eTable 5 in the Supplement). A summary of correlation comparisons for summary PET measures in the CSF TRIAD sample is provided in eTable 1 in the Supplement. A similar pattern of results was observed when examining CSF Aβ42/40 and tau-PET (eResults, eFigure 2, and eTable 2 in the Supplement). The CSF Aβ42/40 ratio was more closely associated with amyloid PET than tau PET (eFigure 3 in the Supplement). In analyses stratified by the presence of cognitive impairment, p-tau biomarkers were much more strongly associated with amyloid PET in CU individuals, whereas no differences were detected in cognitively impaired individuals (eTables 3 and 4 in the Supplement). Analyses comparing amyloid PET and tau PET across the entire cerebral cortex yielded the same pattern of results, although the results were stronger in magnitude (eTable 6 in the Supplement). In analyses comparing global amyloid PET to tau PET in Braak I-II regions, only p-tau_217_ and p-tau_231_ were significantly more closely associated with amyloid PET (eTable 7 in the Supplement). Partial correlation analyses revealed that CSF p-tau biomarkers were more closely associated with amyloid PET when controlling for tau-PET (eTable 8 in the Supplement). The same pattern of results held when controlling for age (eTable 9 in the Supplement) and sex (eTable 10 in the Supplement). Furthermore, CSF p-tau_181_ in ADNI was more closely correlated with summary measurements of amyloid PET than with summary measurements of tau PET (*t* value, 2.21; 95% CI, 0.01-0.21; *P* < .05) (Figure 3). Subgroup analyses are reported in the eTable 11 in the Supplement.

### Associations Between Plasma p-Tau and PET Biomarkers

**Figure 4.**
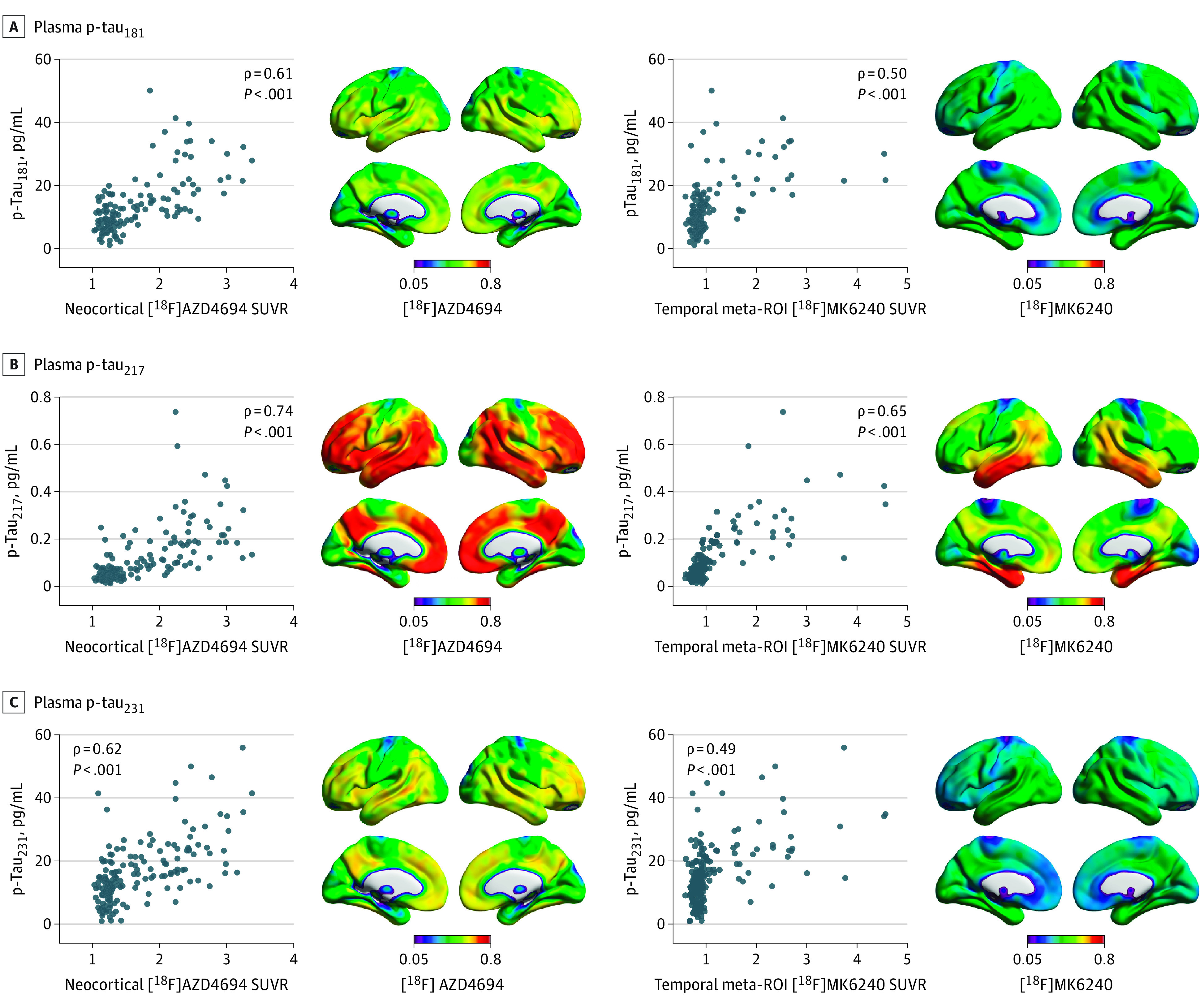
Association of Plasma Phosphorylated Tau (p-Tau) Biomarkers With Amyloid Positron Emission Tomography (PET) and Tau PET Scatterplots show the association between plasma p-tau_181_, p-tau_217_, p-tau_231_, and summary measures of amyloid PET and tau PET in the Translational Biomarkers in Aging and Dementia study. Brain images show the voxelwise associations of plasma p-tau_181_, p-tau_217_, and p-tau_231_ with [^18^F]AZD4694 standardized uptake value ratio (SUVR) and [^18^F]MK6240 SUVR. ROI indicates region of interest.

Finally, we investigated associations of plasma concentrations of p-tau_181_, p-tau_217_, and p-tau_231_ with amyloid PET and tau PET in a nonoverlapping subsample in TRIAD (the individuals in the plasma analyses reported here did not undergo CSF p-tau assessments). Voxelwise analyses revealed that plasma p-tau_181_, p-tau_217_, and p-tau_231_ had strong associations with amyloid PET across the neocortex (Figure 4). Furthermore, p-tau_181_, p-tau_217_, and p-tau_231_ were closely associated with summary measures of amyloid-PET uptake (p-tau_181_, ρ = 0.61; 95% CI, 0.49-0.70; *P* < .001; p-tau_217_, ρ = 0.74; 95% CI, 0.66-0.81; *P* < .001; p-tau_231_, ρ = 0.62; 95% CI, 0.51-0.73; *P* < .001). In comparison, associations between plasma concentrations of p-tau_181_, p-tau_217_, and p-tau_231_ with tau PET were lower (p-tau_181_, ρ = 0.50; 95% CI, 0.39-0.64; *P* < .001; p-tau_217_, ρ = 0.64; 95% CI, 0.54-0.73; *P* < .001; p-tau_231_, ρ = 0.49; 95% CI, 0.37-0.61; *P* < .001), including in medial temporal cortices. Comparison of correlations revealed that p-tau_181_, p-tau_217_, and p-tau_231_ were significantly more closely associated with amyloid PET than with tau PET (plasma p-tau_181_ difference, 11%; 95% CI, 1%-22%; *P* = .02; p-tau_217_ difference, 9%; 95% CI, 1%-19%; *P* = .02; p-tau_231_ difference, 13%; 95% CI, 3%-24%; *P* = .009). A summary of the correlation comparisons in the plasma TRIAD sample is provided in eTable 12 in the Supplement. Partial correlation analyses revealed that plasma p-tau biomarkers were more closely associated with amyloid PET when controlling for tau PET (eTable 13 in the Supplement). Results were similar when controlling for age (eTable 14 in the Supplement).

## Discussion

This cross-sectional study of 2 observational cohorts investigated the association between fluid measures of p-tau with amyloid-β plaques and tau neurofibrillary tangles assessed with PET. For all 4 p-tau phosphorylation sites examined in CSF, p-tau was more closely associated with cerebral amyloid-β plaques than with tau neurofibrillary tangles. These results were replicated with plasma p-tau_181_, p-tau_217_, and p-tau_231_ in an independent subsample and with CSF p-tau_181_ in a large independent cohort of individuals assessed with different amyloid-PET and tau-PET imaging agents. Our findings highlight the need for careful interpretation of p-tau biomarkers in the context of the A/T/(N) biomarker framework and for the biomarker-assisted identification of AD, especially in CU individuals.

Observational studies in humans have reported strong correlations between concentrations of amyloid PET and p-tau at various phosphorylation sites in individuals at different clinical stages of AD.^14,17,28,29,30^ Strong associations between antemortem plasma p-tau_181_, p-tau_217_, and p-tau_231_ with amyloid-β plaques at autopsy have also been reported.^31^ In longitudinal observational studies, CU individuals with elevated amyloid-PET burden had increased concentrations of plasma and CSF p-tau_217_ and p-tau_181_ in the absence of neocortical tau-PET deposition.^16,32,33^ Furthermore, soluble p-tau appeared to drive the association between amyloid-β plaques and insoluble tau aggregation measured with PET.^16,33^ In individuals with dominantly inherited AD, p-tau_217_ closely followed by p-tau_181_ increased in response to amyloid-β accumulation, subsequently followed by tau-PET abnormality several years later.^15^ Furthermore, plasma p-tau_181_ is elevated in individuals who are amyloid-PET positive but tau-PET negative (even in Braak I regions)^32^ and is elevated approximately 16 years before the onset of symptoms in dominantly inherited AD.^34^ Moreover, a study using PET-based Braak staging suggested that both amyloid-PET and p-tau concentrations in CSF plateau at late stages of tangle aggregation,^35^ in agreement with a recent autopsy study.^36^ Taken together, these studies provide converging evidence supporting strong associations between amyloid-β plaques and p-tau biomarkers, which both precede widespread neurofibrillary tangle aggregation.

Several recent preclinical studies have reported that soluble p-tau levels in cell media, human tissue samples, and mouse models rise in response to aggregated amyloid-β.^37,38,39,40^ In vitro models suggest that tau hyperphosphorylation is induced in neurons that take up neuronally secreted amyloid-β.^37,38^ Amyloid-β plaques are also linked to increased neuronal release of hyperphosphorylated tau.^39^ Studies in transgenic mice report that p-tau concentrations in CSF rise as a consequence of amyloid-β deposition,^40^ and human neural stem cell–derived cell culture systems overexpressing *APP* and *PSEN1* induced tau phosphorylation closely linked with amyloid-β concentrations.^41^ A study using stable isotope labeling kinetics in humans demonstrated that soluble p-tau production was positively correlated with amyloid-PET signal but did not change in the presence of elevated tau PET.^42^ These studies support increased tau phosphorylation as an early event in the amyloid-β cascade, closely linked with concentrations of amyloid-β pathology.

Recent p-tau biomarker studies have raised questions about the preferential association of various p-tau epitopes with AD stage, severity, and neuropathological hallmarks.^43^ Tau can be phosphorylated at over 80 different sites on the tau protein,^44^ and the pathophysiological roles of phosphorylation at different sites are unclear.^45^ However, recent studies have provided evidence that specific p-tau phosphorylation sites appear to become elevated in a disease stage-dependent manner^15,46^ and that phosphorylation at specific peptides is associated with increased tau seeding activity and clinical disease progression.^47^ In our study, p-tau biomarkers were most closely associated with tau aggregation in medial temporal brain regions (though still less so than with global amyloid PET), supporting p-tau as an early biomarker. This result is consistent with a recent community-based study that observed a stronger association between plasma p-tau with entorhinal tau PET than commonly used summary measures of tau PET.^48^ Of the 4 p-tau biomarkers examined in CSF, p-tau_217_ and p-tau_231_ showed the highest association with amyloid PET. In plasma, p-tau_217_ was most closely associated with cerebral amyloid-PET concentrations. Although site-specific patterns of tau phosphorylation may provide information regarding disease stage in AD, it also will be crucial to understand the contribution of analytical properties of different assays.

Our study highlights the need for a granular approach to tau biomarkers, in which different tau biomarkers provide complementary but not interchangeable information.^21,28,45,49,50^ Although the tau biomarker category in the A/T/(N) framework currently includes tau PET and p-tau in biofluids,^3,4^ it is important to draw distinctions between both classes of biomarkers. Tau-PET ligands are considered to bind to insoluble neurofibrillary tangles, consisting of paired helical filament aggregates of hyperphosphorylated tau.^51,52^ Fluid biomarkers of soluble p-tau, in contrast, measure the concentration of tau phosphorylated at specific serine, threonine, or tyrosine amino acids on the tau protein, which have leaked from the extracellular space into the CSF or blood compartments. CSF and plasma p-tau biomarkers are reported to rise early in the AD pathophysiological process.^8,16^ Tau tangle aggregation measured with PET occurs later and is strongly predictive of cognitive decline.^53^ In this connection, the much higher association of p-tau biomarkers with amyloidosis than tangle burden in CU individuals suggests that p-tau biomarkers may be less well-positioned to predict future cognitive decline. In contrast, the high association of p-tau biomarkers with both amyloidosis and tangle burden in individuals with cognitive impairment suggest that abnormal p-tau biomarkers have a strong predictive value for AD in diagnostic settings. Taken together, these results highlight the need to distinguish between biomarkers of phosphorylated and aggregated tau in the A/T/(N) framework, particularly in CU individuals.

### Limitations

Results of this study should be interpreted in the context of several limitations. First, tau (hyper)phosphorylation is a dynamic process, the understanding of which is anticipated to evolve with respect to the availability of more biomarkers. Because tau can be phosphorylated at over 80 sites, some of which are hypothesized to have site-specific associations with disease stage, it is unknown whether all future p-tau biomarkers will exhibit the preferential association with amyloid PET reported in this study. Another limitation is that PET biomarker signals (used in this study as measurements of amyloid-β plaques and tau tangles) are influenced by their affinities (1 / equilibrium dissociation constant [K_d_]) for their target. However, because [^18^F]MK6240 has a higher affinity for tau tangles than [^18^F]AZD4694 does for amyloid-β plaques, the stronger association of p-tau with amyloid PET is unlikely to be driven by sensitivity issues. Replication in an independent cohort (ADNI) with different PET imaging agents helps further attenuate these concerns. Similar to PET biomarkers, biofluid assay performance can affect interpretation of the results in this study. For example, it is unclear to what degree the stronger associations of p-tau_217_ and p-tau_231_ with PET biomarkers are driven by biological properties of phosphorylation of a specific amino acid compared with differences in the assays used to detect them (ie, antibody affinity, robustness of individual reagents, assay platform). Other limitations of this study include the lack of availability of plasma p-tau_235_ in the TRIAD cohort and the lack of availability of plasma p-tau_181_ at the same time point as tau-PET in ADNI. Furthermore, the TRIAD and ADNI cohorts consist of individuals motivated to participate in a study of AD, which may limit generalizability. Finally, the cohorts are not demographically representative of the populations at risk for dementia in North America.

## Conclusions

In conclusion, results of this cross-sectional study of 2 observational cohorts suggest that p-tau biomarkers better reflect the concentration of amyloid-β plaques than cerebral tau pathology quantified with PET. Our findings contribute to the growing understanding of the role of tau phosphorylation in the amyloid-β cascade and highlight the need for careful interpretation of p-tau biomarkers in CU individuals and as outcomes in disease-modifying clinical trials.^7^

## Notes

Supplement. **eMethods 1.** Positron Emission Tomography Image Acquisition and Processing **eMethods 2.** CSF and Plasma Sample Handling and Assays **eResults.** Sensitivity Analyses **eFigure 1.** Relationship Between CSF p-Tau Biomarkers and Early PET ROIs **eFigure 2.** Relationship Between CSF p-Tau Biomarkers and CSF Aβ42/40 and Tau-PET **eFigure 3.** Relationship of CSF Aβ42/40 Ratio With Amyloid-PET and Tau-PET **eTable 1.** Statistical Comparison of Correlation Between CSF p-Tau Biomarkers and Summary Amyloid- and Tau-PET Biomarkers in TRIAD **eTable 2.** Statistical Comparison of Correlations Between CSF p-Tau Biomarkers With CSF Aβ42/40 and Tau-PET Biomarkers in TRIAD **eTable 3.** Statistical Comparison of Correlations Between CSF p-Tau With Amyloid-PET and Tau-PET in CU Individuals in TRIAD **eTable 4.** Statistical Comparison of Correlations Between CSF p-Tau With Amyloid-PET and Tau-PET in CI Individuals in TRIAD **eTable 5.** Statistical Comparison of Correlations Between CSF p-Tau Biomarkers and Early Amyloid- and Tau-PET Biomarkers in TRIAD **eTable 6.** Statistical Comparison of Correlations Between CSF p-Tau Biomarkers and Whole-Cortex Amyloid-PET and Whole-Cortex Tau-PET in TRIAD **eTable 7.** Statistical Comparison of Correlations Between CSF p-Tau Biomarkers and Summary Amyloid-PET and Braak I-II Tau-PET Biomarkers in TRIAD **eTable 8.** Statistical Comparison of Partial Correlations Between CSF p-Tau With Amyloid-PET and Tau-PET in TRIAD Correcting for Each Other **eTable 9.** Statistical Comparison of Partial Correlations Between CSF p-Tau With Amyloid-PET and Tau-PET in TRIAD Correcting for Age **eTable 10.** Statistical Comparison of CSF p-Tau Associations With Amyloid-PET and Tau-PET in TRIAD Correcting for Sex **eTable 11.** Statistical Comparison of Correlations Between CSF p-Tau With Amyloid-PET and Tau-PET in ADNI **eTable 12.** Statistical Comparison of Correlations Between Plasma p-Tau Biomarkers and Summary Amyloid-PET and Tau-PET Biomarkers in TRIAD **eTable 13.** Statistical Comparison of Correlations Between Plasma p-Tau Biomarkers With Amyloid-PET and Tau-PET in TRIAD Correcting for Each Other **eTable 14.** Statistical Comparison of Correlations Between Plasma p-Tau Biomarkers With Amyloid-PET and Tau-PET in TRIAD Correcting for Age
References
