# Supplementary material for: Association of Phosphorylated Tau Biomarkers With Amyloid Positron Emission Tomography vs Tau Positron Emission Tomography
Source: JAMA Neurol. 2022 Dec 12;80(2):188–99. doi: 10.1001/jamaneurol.2022.4485 (PMC9856704; doi:10.1001/jamaneurol.2022.4485)
Supplement: Supplement. — eMethods 1. Positron Emission Tomography Image Acquisition and Processing eMethods 2. CSF and Plasma Sample Handling and Assays eResults. Sensitivity Analyses eFigure 1. Relationship Between CSF p-Tau Biomarkers and Early PET ROIs eFigure 2. Relationship Between CSF p-Tau Biomarkers and CSF Aβ42/40 and Tau-PET eFigure 3. Relationship of CSF Aβ42/40 Ratio With Amyloid-PET and Tau-PET eTable 1. Statistical Comparison of Correlation Between CSF p-Tau Biomarkers and Summary Amyloid- and Tau-PET Biomarkers in TRIAD eTable 2. Statistical Comparison of Correlations Between CSF p-Tau Biomarkers With CSF Aβ42/40 and Tau-PET Biomarkers in TRIAD eTable 3. Statistical Comparison of Correlations Between CSF p-Tau With Amyloid-PET and Tau-PET in CU Individuals in TRIAD eTable 4. Statistical Comparison of Correlations Between CSF p-Tau With Amyloid-PET and Tau-PET in CI Individuals in TRIAD eTable 5. Statistical Comparison of Correlations Between CSF p-Tau Biomarkers and Early Amyloid- and Tau-PET Biomarkers in TRIAD eTable 6. Statistical Comparison of Correlations Between CSF p-Tau Biomarkers and Whole-Cortex Amyloid-PET and Whole-Cortex Tau-PET in TRIAD eTable 7. Statistical Comparison of Correlations Between CSF p-Tau Biomarkers and Summary Amyloid-PET and Braak I-II Tau-PET Biomarkers in TRIAD eTable 8. Statistical Comparison of Partial Correlations Between CSF p-Tau With Amyloid-PET and Tau-PET in TRIAD Correcting for Each Other eTable 9. Statistical Comparison of Partial Correlations Between CSF p-Tau With Amyloid-PET and Tau-PET in TRIAD Correcting for Age eTable 10. Statistical Comparison of CSF p-Tau Associations With Amyloid-PET and Tau-PET in TRIAD Correcting for Sex eTable 11. Statistical Comparison of Correlations Between CSF p-Tau With Amyloid-PET and Tau-PET in ADNI eTable 12. Statistical Comparison of Correlations Between Plasma p-Tau Biomarkers and Summary Amyloid-PET and Tau-PET Biomarkers in TRIAD eTable 13. Statistical Comparison of Correlations Between Plasma [file jamaneurol-e224485-s001.pdf]

Supplementary Online Content

Therriault J, Vermeiren M, Servaes S, et al. Association of phosphorylated tau biomarkers with amyloid positron emission tomography vs tau positron emission tomography. *JAMA Neurol*. Published online December 12, 2022. doi:10.1001/jamaneurol.2022.4485

eMethods 1. Positron Emission Tomography Image Acquisition and Processing

eMethods 2. CSF and Plasma Sample Handling and Assays

eResults. Sensitivity Analyses

eFigure 1. Relationship Between CSF p-Tau Biomarkers and Early PET ROIs

eFigure 2. Relationship Between CSF p-Tau Biomarkers and CSF Aβ42/40 and Tau-PET

eFigure 3. Relationship of CSF Aβ42/40 Ratio With Amyloid-PET and Tau-PET

eTable 1. Statistical Comparison of Correlation Between CSF p-Tau Biomarkers and Summary Amyloid- and Tau-PET Biomarkers in TRIAD

eTable 2. Statistical Comparison of Correlations Between CSF p-Tau Biomarkers With CSF Aβ42/40 and Tau-PET Biomarkers in TRIAD

eTable 3. Statistical Comparison of Correlations Between CSF p-Tau With Amyloid-PET and Tau-PET in CU Individuals in TRIAD

eTable 4. Statistical Comparison of Correlations Between CSF p-Tau With Amyloid-PET and Tau-PET in CI Individuals in TRIAD

eTable 5. Statistical Comparison of Correlations Between CSF p-Tau Biomarkers and Early Amyloid- and Tau-PET Biomarkers in TRIAD

eTable 6. Statistical Comparison of Correlations Between CSF p-Tau Biomarkers and Whole-Cortex Amyloid-PET and Whole-Cortex Tau-PET in TRIAD

eTable 7. Statistical Comparison of Correlations Between CSF p-Tau Biomarkers and Summary Amyloid-PET and Braak I-II Tau-PET Biomarkers in TRIAD

eTable 8. Statistical Comparison of Partial Correlations Between CSF p-Tau With Amyloid-PET and Tau-PET in TRIAD Correcting for Each Other

eTable 9. Statistical Comparison of Partial Correlations Between CSF p-Tau With Amyloid-PET and Tau-PET in TRIAD Correcting for Age

eTable 10. Statistical Comparison of CSF p-Tau Associations With Amyloid-PET and Tau-PET in TRIAD Correcting for Sex

eTable 11. Statistical Comparison of Correlations Between CSF p-Tau With Amyloid-PET and Tau-PET in ADNI

© 2022 Therriault J et al. *JAMA Neurology.*

eTable 12. Statistical Comparison of Correlations Between Plasma p-Tau Biomarkers and Summary Amyloid-PET and Tau-PET Biomarkers in TRIAD

eTable 13. Statistical Comparison of Correlations Between Plasma p-Tau Biomarkers With Amyloid-PET and Tau-PET in TRIAD Correcting for Each Other

eTable 14. Statistical Comparison of Correlations Between Plasma p-Tau Biomarkers With Amyloid-PET and Tau-PET in TRIAD Correcting for Age

eReferences

This supplementary material has been provided by the authors to give readers additional information about their work.

© 2022 Therriault J et al. *JAMA Neurology.*

eMethods 1. Positron Emission Tomography Image Acquisition and Processing

TRIAD

[^{18}F]AZD4694 PET and [^{18}F]MK6240 PET scans were acquired with a brain-dedicated Siemens High Resolution Research Tomograph (HRRT). [^{18}F]AZD4694 PET images were acquired 40-70 min after injection of the radiotracer, and reconstructed with an ordered subset expectation maximization algorithm (OSEM) algorithm on a 4-dimensional volume with 3 frames (3 x 600s), as previously described [1]. [^{18}F]MK6240 PET images were acquired at 90-110 min after injection of the radiotracer and reconstructed using an OSEM algorithm on a 4-dimensional volume with 4 frames (4 x 300s) [2]. At the end of each PET acquisition, a 6-min transmission scan with a rotating ^{137}Cs point source was conducted for attenuation correction. PET images were corrected for motion, decay, dead time, random and scattered coincidences. T1-weighted images underwent correction for nonuniformity and field-distortion and were processed using an in-house pipeline. PET images were then automatically registered to the T1-weighted image space, and the T1-weighted images were linearly and non-linearly registered to the MNI reference space. To minimize interference of meningeal spillover into adjacent brain regions, [^{18}F]MK6240 images were meninges-striped in native space before they were transformed and blurred, as described previously [3]. PET linear and non-linear registration to the MNI space was performed using the transformations from the T1-weighted image to MNI space and from the PET image to T1-weighted image space. [^{18}F]AZD4694 standardized uptake value ratio (SUVR) maps were calculated using the whole cerebellum gray matter as the reference region and [^{18}F]MK6240 SUVR maps were generated using the cerebellar crus I matter as a reference region. Spatial smoothing allowed the PET images to achieve an 8-mm full-width at half-maximum resolution.

A composite neocortical amyloid-β region of interest (ROI) SUVR for each participant was estimated by averaging the SUVR from the precuneus, prefrontal, orbitofrontal, parietal, temporal, and cingulate cortices [1]. The SUVR from the temporal meta-ROI, a composite mask commonly used as a summary measure of tau-PET, was calculated from the entorhinal, parahippocampal, amygdala, fusiform, inferior and middle temporal cortices, as previously described [4].

ADNI

Complete details of acquisition of PET data in the ADNI cohort can be accessed at http://adni.loni.usc.edu/data-samples/pet/. [^{18}F]Flortaucipir SUVRs were estimated using the cerebellar gray matter as a reference region and [^{18}F]Florbetapir SUVRs were calculated using the cerebellar gray matter as a reference region. A global [^{18}F]Florbetapir SUVR value was generated for each participant by averaging the SUVR from the precuneus, prefrontal, orbitofrontal, parietal, temporal, anterior, and posterior cingulate cortices [4]. SUVRs from the temporal meta-ROI were calculated from the entorhinal, amygdala, fusiform, inferior and middle temporal cortices, as previously described [4]. Furthermore, whole-cortex ROIs were employed for both amyloid-PET and tau-PET.

eMethods 2. CSF and Plasma Sample Handling and Assays

TRIAD

We assessed p-tau at four phosphorylation sites in CSF: p-tau$_{181}$, p-tau$_{217}$, p-tau$_{231}$, and p-tau$_{235}$. CSF samples were retrieved with lumbar puncture, first using an 18 ga "introducer" to penetrate the interspinous ligaments, followed by dural puncture using the 24 ga Sprotte atraumatic needle. 29 ml of fluid was collected with polypropylene syringes, from which the first 4 mL were sent to a local laboratory for routine analyses. The remaining volume was preserved in polypropylene tubes and centrifuged at 20 degrees Celsius (°C) for 10min at 2200g, after which samples were rapidly frozen for permanent storage at -80°C. All p-tau residues measured from CSF were quantified in the Clinical Neurochemistry Laboratory, University of Gothenburg by scientists blinded to participant clinical information. CSF concentrations of p-tau$_{181}$ and p-tau$_{217}$ were quantified using a custom single molecule array (Simoa) assay. CSF measures of p-tau$_{235}$ were assessed using an in-house developed Simoa assay comprised by a rabbit polyclonal antibody specific for p-tau$_{235}$ conjugated to paramagnetic beads, and mouse monoclonal Tau12 (epitope 6-18aa) as detector [5]. CSF p-tau$_{231}$ was measured using an ELISA assay, as described previously [6]. CSF concentrations of amyloid-β (Aβ40 and Aβ42) were quantified using the fully-automated LUMIPULSE® G1200 instrument (Fujirebio, Ghent, Belgium), using antibody-coated beads for capture, as well

© 2022 Therriault J et al. *JAMA Neurology.*

as monoclonal antibodies for detection. Blood samples were collected following previously described protocols [7]. Plasma p-tau_{181} and plasma p-tau_{231} were also measured in the Clinical Neurochemistry Laboratory, University of Gothenburg by scientists blinded to participant clinical information. Both plasma biomarkers were assessed using an in-house Simoa method (Simoa HD-X instruments, Quanterix, Billerica, MA, USA), as described previously [8,9]. Plasma p-tau_{217} concentrations were measured using a Simoa assay developed by Janssen [10,11]. The assay detects phosphorylation at the threonine amino acid at position 217, enhanced by phosphorylation at the threonine amino acid at position 212.

ADNI

CSF measurements of p-tau_{181} were assessed using the multiplex xMAP Luminex platform (Luminex, Austin, TX, USA) with INNOBIA AlzBio3 (Innogenetics, Ghent, Belgium) immunoassay kit-based reagents. The CSF biomarker data was derived from the ADNI file 'UPENNBIOMK5-8.csv'. Full information on CSF methods used in ADNI can be accessed at http://adni.loni.usc.edu/data-samples/clinical-data/.

eResults. Sensitivity Analyses

Sensitivity analyses

We conducted sensitivity analyses in which we examined summary PET measurements considered to become abnormal earlier in the disease course. eFigure 1 in the supplement shows associations between CSF p-tau biomarkers with early summary measures of amyloid-PET and tau-PET in TRIAD. Of the four phosphorylation sites examined, CSF p-tau_{217} and p-tau_{231} were most closely associated with early amyloid-PET accumulation (p-tau_{217} ρ = 0.77, 95% CI 0.70-0.82, $p<0.001$; p-tau_{231} ρ = 0.79, 95% CI: 0.72-0.84, $p<0.001$). Correlations were lower for p-tau_{181} (ρ = 0.69, 96% CI 0.61-0.77, $p < 0.001$) and p-tau_{235} (ρ = 0.69, 95% CI = 0.60-0.76, $p < 0.001$). When investigating associations with tau-PET in the inferior temporal cortex and CSF p-tau, we observed that p-tau_{217} had the highest coefficient of determination (ρ = 0.62, 95% CI: 0.51-0.70 $p < 0.001$), followed by p-tau_{231}, p-tau_{235} and p-tau_{181}. Comparison of correlations revealed that for all p-tau phosphorylation sites, p-tau was significantly more closely associated with early summary measurements of amyloid-PET than with early measures of tau-PET ($p < 0.01$ for all comparisons). A summary of correlation coefficient comparisons for early summary PET measures in the CSF TRIAD sample is provided in eTable 5. When comparing global amyloid-PET values to very early tau accumulation (Braak I-II regions), p-tau_{217} and p-tau_{231} were still more closely correlated with amyloid-PET, while p-tau_{181} and p-tau_{235} were not statistically different, despite having higher numerical correlation coefficients with amyloid-PET.

We also assessed whether p-tau biomarkers were more closely associated with the CSF Aβ42/40 ratio than tau-PET. Scatterplots for all biomarkers are displayed in eFigure2. CSF p-tau_{217}, CSF p-tau_{231}, and CSF p-tau_{235} were all more closely associated with the CSF Aβ42/40 ratio than tau-PET (eTable2). While CSF p-tau_{181} was numerically more closely associated with the CSF Aβ42/40 ratio than tau-PET, this difference did not reach statistical significance (eTable2).

© 2022 Therriault J et al. *JAMA Neurology.*

eFigure 1. Relationship Between CSF p-Tau Biomarkers and Early PET ROIs

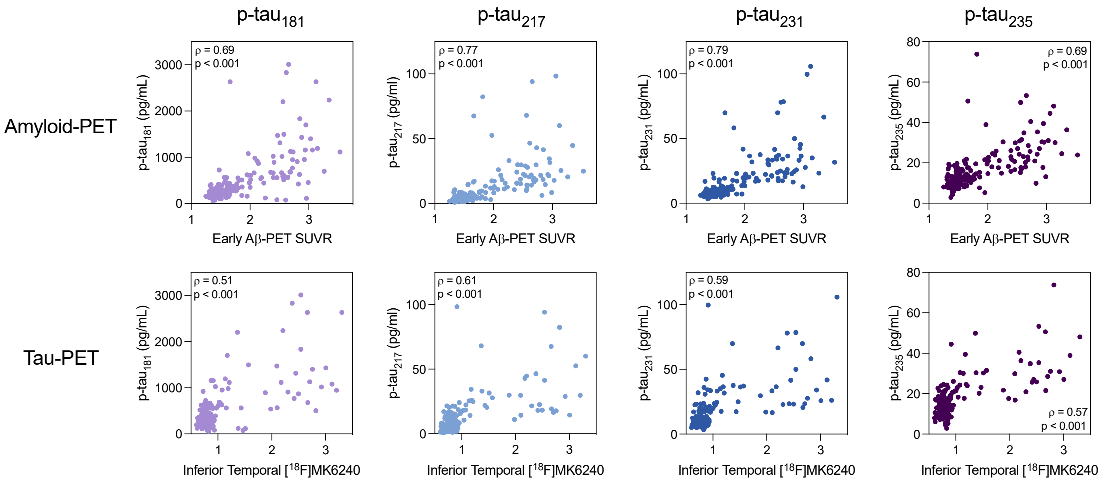

Scatterplots show the relationship between CSF p-tau$_{181}$, p-tau$_{217}$, p-tau$_{231}$, p-tau$_{235}$ and early measures of amyloid-PET and tau-PET in the TRIAD cohort. Corresponding statistics are reported in eTable5.

© 2022 Therriault J et al. *JAMA Neurology.*

eFigure 2. Relationship Between CSF p-Tau Biomarkers and CSF Aβ42/40 and Tau-PET

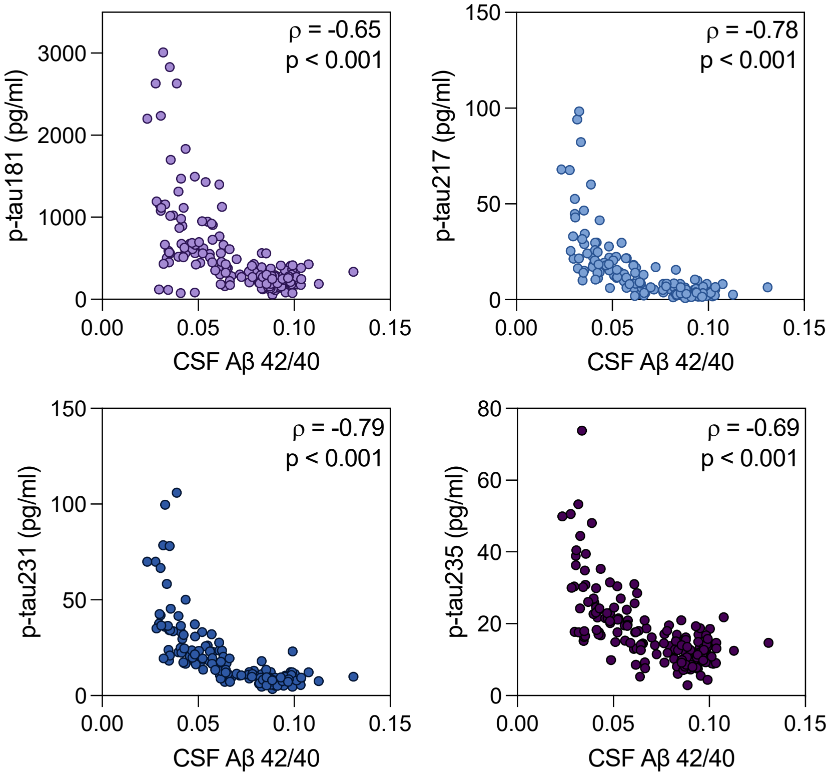

Scatterplots show the relationship between CSF p-tau$_{181}$, p-tau$_{217}$, p-tau$_{231}$, p-tau$_{235}$ and the CSF Aβ42/40 ratio in the TRIAD cohort. Statistical comparisons between CSF Aβ42/40 ratio and tau-PET SUVRs are reported in eTable 2.

© 2022 Therriault J et al. *JAMA Neurology.*

eFigure 3. Relationship of CSF Aβ42/40 Ratio With Amyloid-PET and Tau-PET

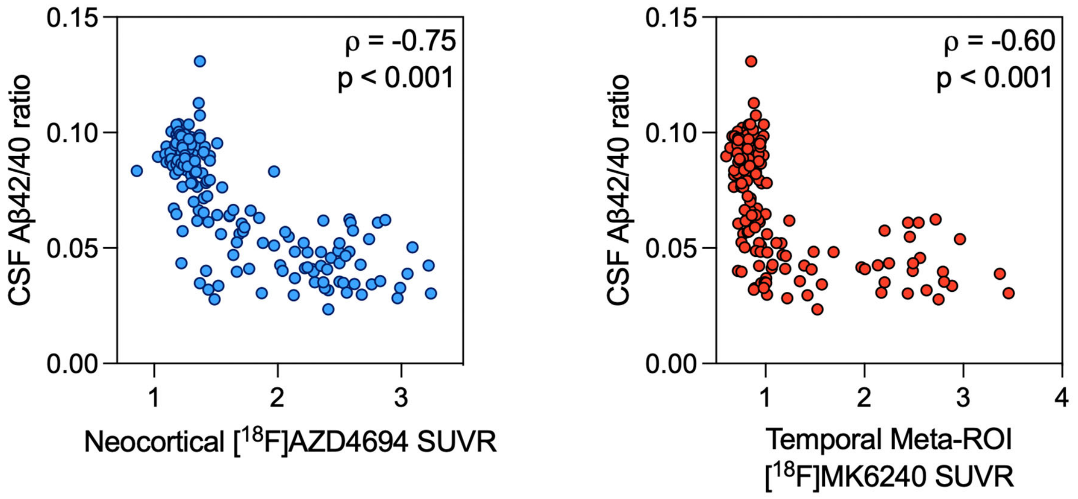

Scatterplots show the relationship between CSF Aβ42/40 ratio with amyloid-PET (left) and tau-PET (right). The CSF Aβ42/40 ratio was significantly more closely associated with amyloid-PET (difference: 0.15, t-value: 3.40, 95% CI: 0.06-0.25, p<0.001)

© 2022 Therriault J et al. *JAMA Neurology.*

eTable 1. Statistical Comparison of Correlation Between CSF p-Tau Biomarkers and Summary Amyloid- and Tau-PET Biomarkers in TRIAD

	Neocortical [^{18}F]AZD4694 SUVR correlation	Temporal meta-ROI [^{18}F]MK6240 SUVR correlation	Comparison T-value	Comparison p-value	95% CI
**CSF p-tau$_{181}$**	0.70	0.57	2.54	0.006	0.03 – 0.22
**CSF p-tau$_{217}$**	0.77	0.66	2.77	0.003	0.03 – 0.20
**CSF p-tau$_{231}$**	0.80	0.65	3.96	<0.001	0.05 – 0.22
**CSF p-tau$_{235}$**	0.70	0.61	1.98	0.02	0.01 – 0.19

P-values and t-values were determined using Hotelling's t test, and confidence intervals were determined using Zhou's test. SUVR = Standardized Uptake Value Ratio.

eTable 2. Statistical Comparison of Correlations Between CSF p-Tau Biomarkers With CSF Aβ42/40 and Tau-PET Biomarkers in TRIAD

	CSF Aβ42/40 correlation	Temporal meta-ROI [^{18}F]MK6240 SUVR correlation	Comparison T-value	Comparison p-value	95% CI
**CSF p-tau$_{181}$**	-0.65	0.57	1.51	0.07	-0.02 – 0.18
**CSF p-tau$_{217}$**	-0.78	0.66	3.01	0.0015	0.04 – 0.21
**CSF p-tau$_{231}$**	-0.79	0.65	3.36	<0.001	0.06 – 0.23
**CSF p-tau$_{235}$**	-0.69	0.61	1.70	0.048	-0.01 – 0.18

P-values and t-values were determined using Hotelling's t test, and confidence intervals were determined using Zhou's test. SUVR = Standardized Uptake Value Ratio. Note that correlation coefficients from CSF Aβ 42/40 and CSF p-tau were changed to a positive sign (multiplied by negative 1) before being compared.

© 2022 Therriault J et al. *JAMA Neurology.*

eTable 3. Statistical Comparison of Correlations Between CSF p-Tau With Amyloid-PET and Tau-PET in CU Individuals in TRIAD

	**Neocortical [^{18}F]AZD4694 SUVR correlation**	**Temporal meta-ROI [^{18}F]MK6240 SUVR correlation**	**Comparison T-value**	**Comparison p-value**	**95% CI**
**CSF p-tau$_{181}$**	0.55	0.27	3.51	<0.001	0.12 – 0.44
**CSF p-tau$_{217}$**	0.59	0.37	2.99	0.002	0.07 – 0.37
**CSF p-tau$_{231}$**	0.63	0.32	4.34	<0.001	0.16 – 0.46
**CSF p-tau$_{235}$**	0.45	0.29	1.95	0.03	0.002 – 0.32

P-values and t-values were determined using Hotelling's t test, and confidence intervals were determined using Zhou's test. SUVR = Standardized Uptake Value Ratio.

eTable 4. Statistical Comparison of Correlations Between CSF p-Tau With Amyloid-PET and Tau-PET in CI Individuals in TRIAD

	**Neocortical [^{18}F]AZD4694 SUVR correlation**	**Temporal meta-ROI [^{18}F]MK6240 SUVR correlation**	**Comparison T-value**	**Comparison p-value**	**95% CI**
**CSF p-tau$_{181}$**	0.62	0.66	-0.47	0.65	-0.21 – 0.13
**CSF p-tau$_{217}$**	0.66	0.71	-0.62	0.54	-0.21 – 0.11
**CSF p-tau$_{231}$**	0.66	0.68	-0.24	0.81	-0.18 – 0.15
**CSF p-tau$_{235}$**	0.62	0.73	-1.42	0.18	-0.29 – 0.05

P-values and t-values were determined using Hotelling's t test, and confidence intervals were determined using Zhou's test. SUVR = Standardized Uptake Value Ratio.

© 2022 Therriault J et al. *JAMA Neurology.*

eTable 5. Statistical Comparison of Correlations Between CSF p-Tau Biomarkers and Early Amyloid- and Tau-PET Biomarkers in TRIAD

	BioFINDER Early Aβ-PET correlation	Inferior temporal [^{18}F]MK6240 SUVR correlation	Comparison T-value	Comparison p-value	95% CI
**CSF p-tau$_{181}$**	0.69	0.51	3.61	<0.001	0.07 – 0.29
**CSF p-tau$_{217}$**	0.77	0.61	3.79	<0.001	0.07 – 0.26
**CSF p-tau$_{231}$**	0.79	0.59	4.86	<0.001	0.11 – 0.30
**CSF p-tau$_{235}$**	0.69	0.57	2.48	0.008	0.02 – 0.22

P-values and t-values were determined using Hotelling's t test, and confidence intervals were determined using Zhou's test. SUVR = Standardized Uptake Value Ratio.

eTable 6. Statistical Comparison of Correlations Between CSF p-Tau Biomarkers and Whole-Cortex Amyloid-PET and Whole-Cortex Tau-PET in TRIAD

	Whole-cortex [^{18}F]AZD4694 SUVR correlation	Whole-cortex [^{18}F]MK6240 SUVR correlation	Comparison T-value	Comparison p-value	95% CI
**CSF p-tau$_{181}$**	0.67	0.43	4.34	<0.001	0.12 – 0.36
**CSF p-tau$_{217}$**	0.74	0.51	4.68	<0.001	0.13 – 0.34
**CSF p-tau$_{231}$**	0.76	0.52	4.46	<0.001	0.13 – 0.35
**CSF p-tau$_{235}$**	0.67	0.50	3.15	0.001	0.06 – 0.27

P-values and t-values were determined using Hotelling's t test, and confidence intervals were determined using Zhou's test. SUVR = Standardized Uptake Value Ratio.

© 2022 Therriault J et al. *JAMA Neurology.*

eTable 7. Statistical Comparison of Correlations Between CSF p-Tau Biomarkers and Summary Amyloid-PET and Braak I-II Tau-PET Biomarkers in TRIAD

	Neocortical [^{18}F]AZD4694 SUVR correlation	Braak I-II [^{18}F]MK6240 SUVR correlation	Comparison T-value	Comparison p-value	95% CI
**CSF p-tau$_{181}$**	0.70	0.66	0.77	0.21	-0.04 – 0.10
**CSF p-tau$_{217}$**	0.77	0.71	1.77	0.04	0.01 – 0.13
**CSF p-tau$_{231}$**	0.80	0.73	2.21	0.02	0.05 – 0.22
**CSF p-tau$_{235}$**	0.70	0.68	0.53	0.29	-0.05 – 0.10

P-values and t-values were determined using Hotelling's t test, and confidence intervals were determined using Zhou's test. SUVR = Standardized Uptake Value Ratio.

eTable 8. Statistical Comparison of Partial Correlations Between CSF p-Tau With Amyloid-PET and Tau-PET in TRIAD Correcting for Each Other

	Neocortical [^{18}F]AZD4694 SUVR correlation	Temporal meta-ROI [^{18}F]MK6240 SUVR correlation	Comparison T-value	Comparison p-value	95% CI
**CSF p-tau$_{181}$**	0.47	0.31	2.61	0.004	0.04 – 0.28
**CSF p-tau$_{217}$**	0.58	0.42	2.84	0.002	0.05 – 0.27
**CSF p-tau$_{231}$**	0.62	0.40	3.95	<0.001	0.11 – 0.33
**CSF p-tau$_{235}$**	0.42	0.39	0.49	0.31	-0.08 – 0.15

P-values and t-values were determined using Hotelling's t test, and confidence intervals were determined using Zhou's test. SUVR = Standardized Uptake Value Ratio.

© 2022 Therriault J et al. *JAMA Neurology.*

eTable 9. Statistical Comparison of Partial Correlations Between CSF p-Tau With Amyloid-PET and Tau-PET in TRIAD Correcting for Age

	**Neocortical [^{18}F]AZD4694 SUVR correlation**	**Temporal meta-ROI [^{18}F]MK6240 SUVR correlation**	**Comparison T-value**	**Comparison p-value**	**95% CI**
**CSF p-tau$_{181}$**	0.66	0.58	1.76	0.04	-0.01 – 0.17
**CSF p-tau$_{217}$**	0.76	0.67	2.36	0.01	0.01 – 0.17
**CSF p-tau$_{231}$**	0.77	0.68	2.31	0.01	0.01 – 0.17
**CSF p-tau$_{235}$**	0.66	0.62	0.90	0.19	-0.05 – 0.13

P-values and t-values were determined using Hotelling's t test, and confidence intervals were determined using Zhou's test. SUVR = Standardized Uptake Value Ratio.

© 2022 Therriault J et al. *JAMA Neurology.*

eTable 10. Statistical Comparison of CSF p-Tau Associations With Amyloid-PET and Tau-PET in TRIAD Correcting for Sex

CSF (TRIAD)	181 estimate (95% CI)	t-val	p-val	217 estimate (95% CI)	t-val	p-val	231 estimate (95% CI)	t-val	p val	235 estimate (95% CI)	t-val	p-val
**Amyloid-PET**	0.29 (0.21-0.35)	7.68	<0.001	0.42 (0.34-0.51)	9.99	<0.001	0.34 (0.28-0.39)	12.1	<0.001	0.17 (0.12-0.21)	6.73	<0.001
**Tau-PET**	0.20 (0.13-0.27)	5.73	<0.001	0.23 (0.15-0.31)	5.78	<0.001	0.15 (0.10-0.20)	5.78	<0.001	0.15 (0.10-0.20)	6.52	<0.001
**Sex**	0.03 (-0.04-0.09)	0.78	0.44	0.03 (-0.05-0.11)	0.66	0.51	0.00 (-0.05-0.05)	0.01	0.99	0.01 (-0.04-0.05)	0.33	0.74

Plasma (TRIAD)	181 estimate (95% CI)	t-val	p-val	217 estimate (95% CI)	t-val	p-val	231 estimate (95% CI)	t-val	p val
**Amyloid-PET**	0.26 (0.17-0.34)	5.86	<0.001	0.30 (0.23-0.37)	8.19	<0.001	0.22 (0.13-0.31)	4.92	<0.001
**Tau-PET**	0.07 (0.00-0.14)	1.96	0.05	0.20 (0.14-0.26)	6.74	<0.001	0.07 (0.00-0.13)	1.98	0.05
**Sex**	0.04 (-0.03-0.13)	1.20	0.23	0.05 (-0.02-0.12)	1.43	0.51	0.02 (-0.07-0.11)	0.40	0.99

CSF (ADNI)	181 estimate (95%CI)	t-val	p-val
**Amyloid-PET**	0.40 (0.29-0.50)	7.33	<0.001
**Tau-PET**	0.16 (0.08-0.25)	3.86	<0.001
**Sex**	-0.03 (-0.07-0.01)	-1.38	0.17

Sex was not associated with p-tau biomarker concentrations and p-tau remained more closely associated with amyloid-PET when including sex as a covariate.

© 2022 Therriault J et al. *JAMA Neurology.*

eTable 11. Statistical Comparison of Correlations Between CSF p-Tau With Amyloid-PET and Tau-PET in ADNI

	Neocortical [^{18}F]Florbetapir correlation	Temporal meta-ROI [^{18}F]Flortaucipir correlation	Comparison T-value	Comparison p-value	95% CI
**CSF p-tau$_{181}$**	0.54	0.43	2.12	0.02	0.01 – 0.21

P-values and t-values were determined using Hotelling's t test, and confidence intervals were determined using Zhou's test. SUVR = Standardized Uptake Value Ratio.

Subgroup analysis of CU individuals in ADNI.

	Neocortical [^{18}F]Florbetapir correlation	Temporal meta-ROI [^{18}F]Flortaucipir correlation	Comparison T-value	Comparison p-value	95% CI
**CSF p-tau$_{181}$**	0.52	0.35	2.19	0.01	0.02 – 0.32

P-values and t-values were determined using Hotelling's t test, and confidence intervals were determined using Zhou's test. SUVR = Standardized Uptake Value Ratio.

Subgroup analysis of CI individuals in ADNI.

	Neocortical [^{18}F]Florbetapir correlation	Temporal meta-ROI [^{18}F]Flortaucipir correlation	Comparison T-value	Comparison p-value	95% CI
**CSF p-tau$_{181}$**	0.50	0.51	-0.14	0.55	-0.15 – 0.14

P-values and t-values were determined using Hotelling's t test, and confidence intervals were determined using Zhou's test. SUVR = Standardized Uptake Value Ratio.

© 2022 Therriault J et al. *JAMA Neurology.*

eTable 12. Statistical Comparison of Correlations Between Plasma p-Tau Biomarkers and Summary Amyloid-PET and Tau-PET Biomarkers in TRIAD

	**Neocortical [18F]AZD4694 SUVR correlation**	**Temporal Meta-ROI [18F]MK6240 SUVR correlation**	**Comparison T-value**	**Comparison p-value**	**95% CI**
**Plasma p-tau$_{181}$**	0.61	0.50	2.04	0.02	0.01 - 0.22
**Plasma p-tau$_{217}$**	0.74	0.65	2.29	0.02	0.01 – 0.19
**Plasma p-tau$_{231}$**	0.62	0.49	2.43	0.009	0.03 – 0.24

P-values and t-values were determined using Hotelling's t test, and confidence intervals were determined using Zhou's test. SUVR = Standardized Uptake Value Ratio.

eTable 13. Statistical Comparison of Correlations Between Plasma p-Tau Biomarkers With Amyloid-PET and Tau-PET in TRIAD Correcting for Each Other

	**Neocortical [18F]AZD4694 SUVR correlation**	**Temporal Meta-ROI [18F]MK6240 SUVR correlation**	**Comparison T-value**	**Comparison p-value**	**95% CI**
**Plasma p-tau$_{181}$**	0.48	0.23	3.92	<0.001	0.12 - 0.38
**Plasma p-tau$_{217}$**	0.55	0.35	3.31	<0.001	0.08 – 0.32
**Plasma p-tau$_{231}$**	0.45	0.18	4.15	<0.001	0.14 – 0.39

P-values and t-values were determined using Hotelling's t test, and confidence intervals were determined using Zhou's test. SUVR = Standardized Uptake Value Ratio.

© 2022 Therriault J et al. *JAMA Neurology.*

eTable 14. Statistical Comparison of Correlations Between Plasma p-Tau Biomarkers With Amyloid-PET and Tau-PET in TRIAD Correcting for Age

	**Neocortical [^{18}F]AZD4694 SUVR correlation**	**Temporal Meta-ROI [^{18}F]MK6240 SUVR correlation**	**Comparison T-value**	**Comparison p-value**	**95% CI**
**Plasma p-tau$_{181}$**	0.61	0.49	2.21	0.01	0.02 - 0.23
**Plasma p-tau$_{217}$**	0.73	0.64	2.04	0.02	0.001 – 0.19
**Plasma p-tau$_{231}$**	0.58	0.48	1.81	0.04	-0.01 – 0.21

P-values and t-values were determined using Hotelling's t test, and confidence intervals were determined using Zhou's test. SUVR = Standardized Uptake Value Ratio.

© 2022 Therriault J et al. *JAMA Neurology.*

eReferences

1. Therriault J, Benedet AL, Pascoal TA, et al. Determining amyloid-β positivity using 18F-AZD4694 PET imaging. *J Nucl Med*. 2021;62(2):247-252. doi:10.2967/jnumed.120.245209
2. Therriault J, Pascoal TA, Benedet AL, et al. Frequency of biologically-defined AD in relation to age, sex, APOEε4 and cognitive impairment. *Neurology*. 2021.
3. Pascoal TA, Therriault J, Benedet AL, et al. 18F-MK-6240 PET for early and late detection of neurofibrillary tangles. *Brain*. 2020;143(9):2818-2830. doi:10.1093/brain/awaa180
4. Jack CR, Wiste HJ, Weigand SD, et al. Defining imaging biomarker cut points for brain aging and Alzheimer's disease. *Alzheimer's Dement*. 2017;13(3):205-216. doi:10.1016/j.jalz.2016.08.005
5. Lantero-Rodriguez J, Snellman A, Benedet AL, et al. P-tau235: a novel biomarker for staging preclinical Alzheimer's disease. *EMBO Mol Med*. 2021;13(12):1-16. doi:10.15252/emmm.202115098
6. Ashton NJ, Benedet AL, Pascoal TA, et al. Cerebrospinal fluid p-tau231 as an early indicator of emerging pathology in Alzheimer's disease. *eBioMedicine*. 2022;76:103836. doi:10.1016/j.ebiom.2022.103836
7. Karikari TK, Pascoal TA, Ashton NJ, et al. Blood phosphorylated tau 181 as a biomarker for Alzheimer's disease: a diagnostic performance and prediction modelling study using data from four prospective cohorts. *Lancet Neurol*. 2020;19(5):422-433. doi:10.1016/S1474-4422(20)30071-5
8. Ashton NJ, Pascoal TA, Karikari TK, et al. Plasma p-tau231: a new biomarker for incipient Alzheimer's disease pathology. *Acta Neuropathol*. 2021;(0123456789). doi:10.1007/s00401-021-02275-6
9. Therriault J, Benedet AL, et al. Association of plasma P-tau181 with memory decline in non-demented adults. *Brain Commun*. 2021;3(3):1-10. doi:10.1093/braincomms/fcab136
10. Triana-Baltzer G, Moughadam S, Slemmon R, et al. Development and validation of a high-sensitivity assay for measuring p217+tau in plasma. *Alzheimer's Dement Diagnosis, Assess Dis Monit*. 2021;13(1):1-14. doi:10.1002/dad2.12204
11. Doré V, Doecke JD, Saad ZS, et al. Plasma p217+tau versus NAV4694 amyloid and MK6240 tau PET across the Alzheimer's continuum. *Alzheimer's Dement Diagnosis, Assess Dis Monit*. 2022;14(1):1-11. doi:10.1002/dad2.12307

© 2022 Therriault J et al. *JAMA Neurology.*
